# The Circadian Clock Gates the Intestinal Stem Cell Regenerative State

**DOI:** 10.1016/j.celrep.2013.03.016

**Published:** 2013-04-11

**Authors:** Phillip Karpowicz, Yong Zhang, John B. Hogenesch, Patrick Emery, Norbert Perrimon

**Affiliations:** 1 Department of Genetics, Harvard Medical School, Boston, MA 02115, USA; 2 Department of Neurobiology, University of Massachusetts Medical School, Worcester, MA 01605, USA; 3 Howard Hughes Medical Institute, Chevy Chase, MD 20815, USA; 4 Department of Pharmacology, University of Pennsylvania School of Medicine, Philadelphia, PA 19104, USA

## Abstract

The intestine has evolved under constant environmental stresses, because an animal may ingest harmful pathogens or chemicals at any time during its lifespan. Following damage, intestinal stem cells (ISCs) regenerate the intestine by proliferating to replace dying cells. ISCs from diverse animals are remarkably similar, and the Wnt, Notch, and Hippo signaling pathways, important regulators of mammalian ISCs, are conserved from flies to humans. Unexpectedly, we identified the transcription factor period, a component of the circadian clock, to be critical for regeneration, which itself follows a circadian rhythm. We discovered hundreds of transcripts that are regulated by the clock during intestinal regeneration, including components of stress response and regeneration pathways. Disruption of clock components leads to arrhythmic ISC divisions, revealing their underappreciated role in the healing process.

## INTRODUCTION

Although many pathways that are required for healing have been discovered, little is known about how or whether healing is synchronized with general processes that regulate an animal’s homeostasis and behavior. The circadian clock is an ancient molecular pathway that synchronizes organisms with daily environmental cues (zeitgebers) such as light intensity and temperature oscillations (Borgs et al., 2009; Hardin, 2011). Circadian rhythms are repeated over a 24 hr cycle, yet this chronological aspect of cell state has received little attention in the field of regenerative biology. For instance, many of the pathways that regulate intestinal regeneration and intestinal stem cells (ISCs) have been the subject of important studies (Biteau et al., 2011;Casali and Batlle, 2009), but most of these studies did not consider whether results obtained during one part of the day occur at all times.

Circadian rhythms are thought to influence the cell cycle (Borgs et al., 2009), and there is some evidence that the clock plays a role in regeneration and proliferation. Hepatocyte cell division exhibits rhythms and is delayed following hepatectomy if circadian rhythms are disrupted (Matsuo et al., 2003). Earlier studies in the intestine indeed found a daily rhythmicity in cell number and villus length (Qiu et al., 1994; Stevenson et al., 1979), as well as proliferation (Al-Nafussi and Wright, 1982;Potten et al., 1977), although clock mutants were not examined and ISCs were not specifically identified in those reports. Further, it was reported that metabolic processes display time-of-day variation in the intestine (Pan and Hussain, 2009; Saito et al., 1976; Scheving, 2000), and *per* mutation hastens tumorigenesis in Wnt pathway-driven colorectal cancer in mice (Wood et al., 2008). Finally, the degree of intestinal mucositis displays time-of-day variability in cancer patients treated by radiation (Shukla et al., 2010). This suggests that circadian rhythms may influence the intestine’s regenerative response, although the reasons for this remain a mystery.

## RESULTS

### The *Drosophila* Intestine Has a Circadian Clock

**Figure 1:**
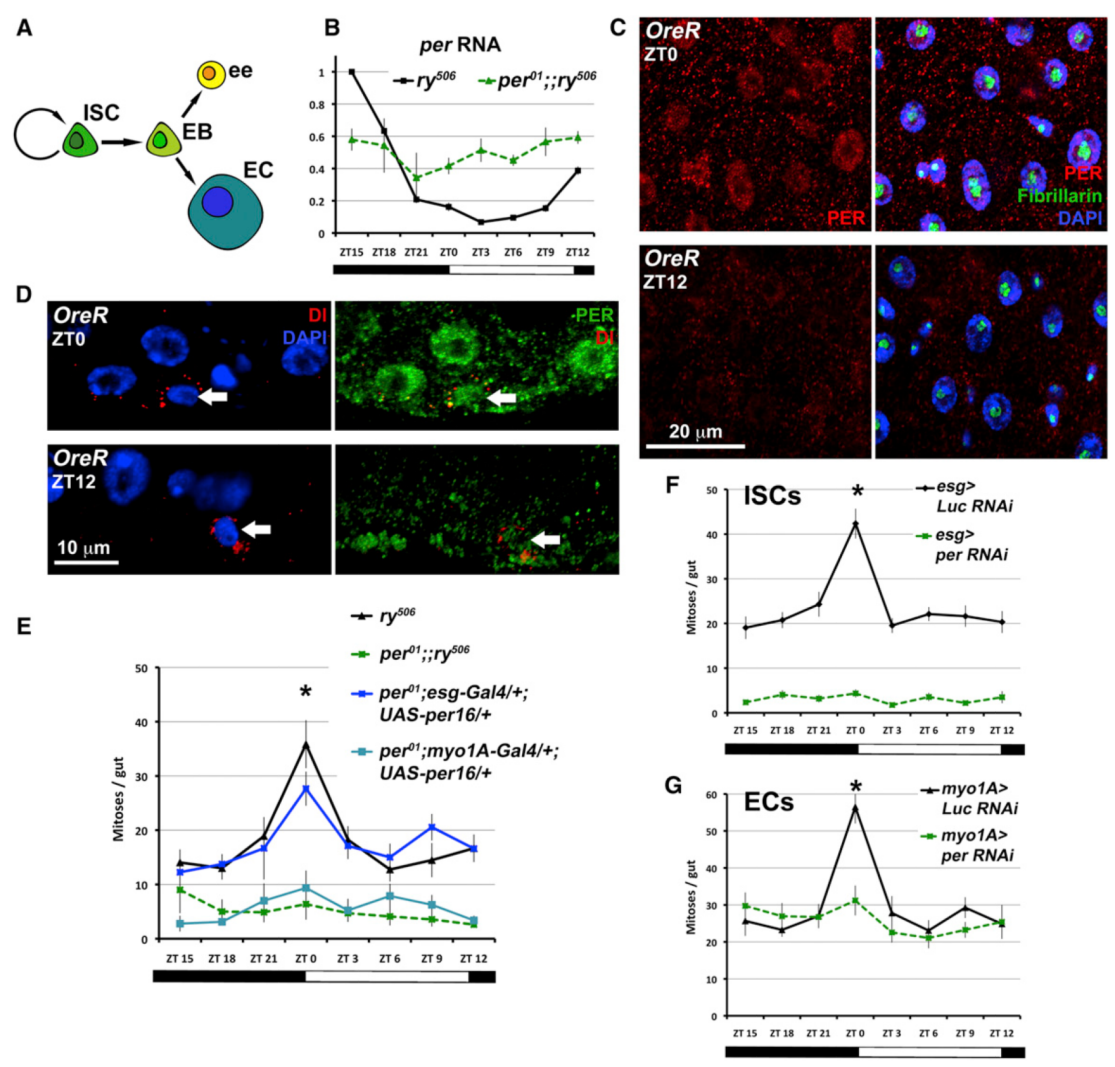
PER Cycles and Functions in the Damaged Intestine (A) The ISC lineage. ISC, intestinal stem cell; EB, enteroblast; ee, enteroendocrine cell; EC, enterocyte. (B) *per* RNA expression (qPCR) in the intestine over ZT, with ZT0 denoting when lights are turned on. The *ry^506^* control normally shows circadian rhythms, but these are absent in *per^01^* mutants. Graph shows the average of two separate experiments (n = 15 guts/genotype/time point, expression normalized to *ry^506^* ZT15, relative to *GAPDH* RNA; error bars ± SEM). (C) PER staining (red) shows nuclear accumulation in intestinal cells in the morning (ZT0) versus the evening (ZT12). Fibrillarin (green) marks the nucleolus, where PER is weaker. (D) PER protein levels are rhythmic in ISCs (arrows) labeled with Delta (Dl, red). (E) When flies are maintained in LD conditions (see Figure S1C for schematic), control (*ry^506^*) intestinal mitoses peak at ZT0, in contrast to *per^01^*. A *UAS-per* rescue construct expressed in ISCs using *esg-Gal4* rescues this effect partially in the *per^01^* background. (F) Rhythms are present in Luciferase (*esg > Luc RNAi* is *esg-Gal4/+; UAS-dcr2/UAS-Luc RNAi*) controls, but PER knockdown in ISCs (*esg > per RNAi* is *esg-Gal4/+; UAS-dcr2/UAS-per RNAi*) phenocopies *per^01^*. (G) PER knockdown in ECs also disrupts circadian mitotic rhythms (genotypes as above but with *myo1A-Gal4/+*). See also Figures S1, S2, S3, and S5.

The intestinal biology of *Drosophila* parallels that of mammals (Biteau et al., 2011; Casali and Batlle, 2009) and allows for functional in vivo analyses to elucidate regenerative processes. *Drosophila* ISCs divide to produce progenitors called enteroblasts (EBs) that differentiate directly into absorptive enterocytes (ECs) or secretory enteroendocrine cells (Figure 1A). We performed a transgenic RNAi screen for transcription factors required in *Drosophila* ISCs during regeneration (see Experimental Procedures). It was previously shown that after damage occurs, ISCs regenerate the intestine by proliferating to replace dying cells (Biteau et al., 2011; Medema and Vermeulen, 2011). Here we discovered that among the ~600 genes tested, *period* (*per*) was required for proliferation of adult ISCs following damage by dextran-sodium sulfate (DSS), a chemical that models inflammatory bowel diseases in flies and mice (Amcheslavsky et al., 2009).

The *Drosophila* circadian pacemaker comprises the transcription factor partners *clock* (*clk*) and *cycle* (*cyc*), which are negatively regulated by *per* and *timeless* (*tim*; Hardin, 2011). One transcriptional target of CLK/CYC is *per* itself, which represses its own production and causes the cyclical transcriptional rhythms that underlie circadian rhythms. The existence of independent clocks throughout *Drosophila* tissues is known (Plautz et al., 1997), and we confirmed the cyclical accumulation and loss of *per* in the intestine when flies were kept on a 12 hr light/12 hr dark (LD) regimen (all of the experiments described below were performed under LD and chemical damage unless otherwise noted). Quantitative RT-PCR (qRT-PCR) confirmed that *per* mRNA accumulates in the early evening (zeitgeber time 12–18 [ZT12–18]; Figures 1B and S1A), and staining for PER confirmed its nuclear accumulation in the late night/early morning (Figure 1C, ZT0). PER is expressed in the epithelial cells of this tissue (the polyploid ECs as well as the diploid ISCs; Figures 1D and S1).

### The Clock Gene *per* Regulates Rhythmic Intestinal Regeneration

The *per^01^* allele is a loss-of-function nonsense mutation (Hardin et al., 1990). Although they are viable, *per^01^* mutant animals do not exhibit circadian gene expression or behavioral rhythmicity (Figures 1B, S1, and S2). We assayed the regenerative response of *per^01^* ISCs following damage by DSS. Only the ISCs in the *Drosophila* intestine divide (Ohlstein and Spradling, 2006), and mitotic ISCs were scored by phosphorylated histone H3 positivity. Control (*ry^506^*) ISCs show a peak in mitoses occurring at dawn (Figure 1E, ZT0), the transition between night and day when PER accumulates. This peak is absent in *per^01^* intestines, which show reduced mitoses at all time points (Figure 1E). A *UAS-per* transgene, which restores circadian rhythms behaviorally when expressed in pacemaker neurons (Figure S2), partially restored the mitotic peak in *per^01^* when expressed in ISCs using *esg-Gal4* (Amcheslavsky et al., 2009), but not in ECs using *myo1A-Gal4* (Jiang et al., 2009; Figure 1E). Importantly, the *esg-Gal4* and *myo1A-Gal4* drivers are not expressed in pacemaker neurons, and do not rescue *per^01^* arrhythmic behavior when driving *UAS-per* (Figure S2). A characteristic of circadian rhythms is their free-running nature (Hardin, 2011), which we tested by shifting flies to constant darkness (DD) after LD entrainment. PER expression rhythms and intestinal mitotic rhythms perpetuate in DD, demonstrating their circadian nature (see Figures S1F and S5A–S5C). Together, these results show that ISCs divide according to a circadian rhythm in response to damage, and that this response is *per* dependent.

Undamaged *per^01^* intestines do not show obvious deficiencies in epithelial cell types (Figures S3A and S3B) or rhythmic mitoses (see Figure 4C). Both ISCs and ECs participate in regeneration (Biteau et al., 2011), raising the question as to which cells are responsible for the inability of *per^01^* intestines to display mitotic rhythms. A second important question is whether mitotic rhythms in response to damage are linked to behavioral activity or feeding (Xu et al., 2008). We validated a *UAS-per RNAi* construct for its ability to reduce PER expression and abolish circadian behavior rhythms (Figure S2). PER knockdown in ISCs phenocopied the arrhythmic *per^01^* intestine (Figure 1F) and, strikingly, PER depletion in ECs also abolished ISC proliferation rhythms (Figure 1G). These phenotypes are not correlated with circadian behavior (Figure S2) or feeding (Figures S3C and S3D), which are rhythmic (although we do note an 1 hr circadian period lengthening in the *esg-Gal4* driver). Since ~ only ISCs divide in this tissue, *per RNAi* disruption in ISCs (Figure 1F) accounts for the *per^01^* phenotype (Figure 1E), whereas *per RNAi* in ECs simply abolishes a peak at ZT0 (Figure 1G). These results suggest that PER is required separately in both ISCs and ECs to produce intestinal mitotic rhythms, and that these rhythms are separate from feeding and behavioral rhythms.

Next, we generated *per*-deficient mutant clones to test whether the defect associated with PER loss was cell autonomous. Following damage, *per^01^* and *per RNAi* clones are slightly smaller (Figure S4) and show reduced size over long periods of time in the absence of acute damage. This suggests that PER has a weaker ISC-autonomous role in initiating or boosting proliferation following damage or stress, but that overall a stronger nonautonomous role is predominant.

### The Core Clock Functions during Intestinal Regeneration

**Figure 2:**
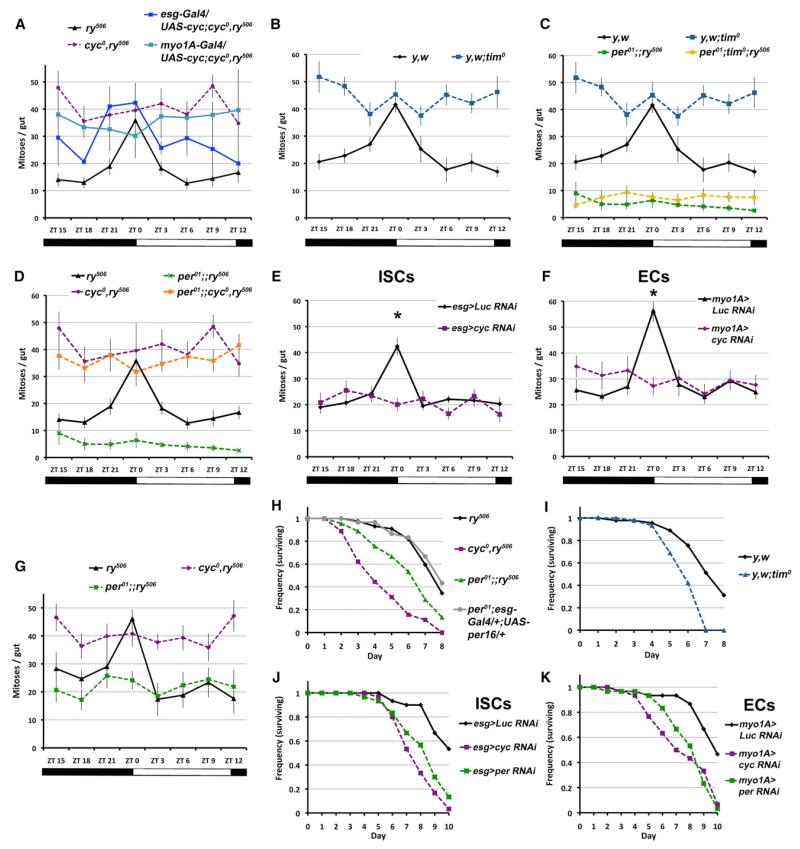
The Circadian Clock Is Required in the Damaged Intestine (A and B) When flies are maintained in LD conditions, control (*ry^506^* and *y,w*) intestinal mitoses peak at ZT0, in contrast to *cyc^0^* and *tim^0^* mutants. A *UAS-cyc* construct expressed in ISCs (*esg-Gal4*) partially restores this rhythm in the *cyc^0^* background. *ry^506^* data are duplicated from Figure 1E. (C and D) *per^01^*; *tim^0^* double-mutant intestines resemble the *per^01^* mutant phenotype. *per^01^*; *cyc^0^* double-mutant intestines resemble the *cyc^0^* mutant phenotype. Control and mutant data are duplicated from Figures 1E, 2A, and 2B. (E and F) CYC knockdown in ISCs (*esg > cyc RNAi* is *esg-Gal4/+; UAS-dcr2/UAS-cyc RNAi*) or in ECs (*myo1A > cyc RNAi* is *myo1A-Gal4/+; UAS-dcr2/UAS-cyc RNAi*) disrupts circadian mitotic rhythms. Control data are from Figures 1F and 1G. All graphs show the average of two separate experiments (n = 10 guts/genotype/time point, error bars ± SEM, *p < 0.05 at ZT0). (G) Following Bleocin exposure, control (*ry^506^*) intestinal mitoses peak at ZT0, in contrast to *per^01^* and *cyc^0^*, similarly to what happens following DSS damage. (H and K) The survival rates of all circadian clock mutants as well as animals in which PER or CYC was knocked down by RNAi in either ISCs or ECs are reduced compared with controls on Bleocin (black lines). Graphs show representative experiments (n = 3 vials, 15 flies per vial; genotypes as above). See also Figures S1, S2, S3, and S5.

Because *per* and *tim* work together to inhibit *clk*/*cyc*, the outcomes of CYC activity would be expected to oppose those of PER. The *cyc^0^* and *tim^0^* loss-of-function mutants are also viable, and also display altered intestinal mitotic rhythms in response to damage (Figures 2A and 2B). The expression of a *UAS-cyc* transgene in ISCs (*esg-Gal4*) in the *cyc^0^* background was able to partially rescue this phenotype, but expression in ECs (*myo1A-Gal4*) did not (Figure 2A). Although the *cyc^0^* phenotype is the opposite of the *per^01^* phenotype, we note that the *tim^0^* phenotype is not the same as that of *per^01^*, suggesting that *tim* may have additional functions in this tissue. It is also possible that genetic background plays a role in the level of mitoses observed in these conditions. We tested the epistatic relationships between these genes. The *per^01^*;*tim^0^* double mutant displays the *per^01^* phenotype (Figure 2C), and the *cyc^0^*;*per^01^* double mutant displays the *cyc^0^* phenotype (Figure 2D), as would be predicted from the circadian clock transcriptional feedback loop, which undergoes circadian rhythms in this tissue (Figure S1). We further tested the requirement of CYC in the regenerative process by expressing a functionally validated *UAS-cyc RNAi* construct (Figures S2 and S3) in ISCs and ECs. CYC is required in both of these cell types to produce mitotic rhythms, and the loss of CYC in either ISCs (Figure 2E) or ECs (Figure 2F) abolished any rhythms observed. Light levels entrain the circadian clock, and when flies are exposed to light-only (LL) conditions, the rhythmic nature of mitoses is abolished and remains constant at all time points (Figures S5E–S5G). Altogether, these data confirm that the circadian clock is required in both ISCs and their EC neighbors for mitotic rhythms.

Bleocin is a potent DNA-damaging chemical that causes apoptosis in the intestine (Amcheslavsky et al., 2009), and it was applied to investigate the outcome of a circadian-deficient damage response. Following Bleocin-induced damage, mitoses in control versus *cyc^0^* and *per^01^* mutant flies show phenotypes similar to those observed under DSS (Figure 2G). The *cyc^0^* mutants exhibit reduced survival on Bleocin (Figure 2H) or DSS (Figure S5), and *per^01^* and *tim^0^* show similar reduced survival (Figures 2H, 2I, and S5). The knockdown of CYC or PER within ISCs or ECs results in reduced survival on Bleocin (Figures 2J and 2K). Hence, the disruption of the circadian clock either throughout the body or only in ISCs or ECs negatively impacts the survival of animals when the intestine is damaged.

### Clock-Deficient ISCs Lag in the Cell Cycle during Regeneration

**Figure 3:**
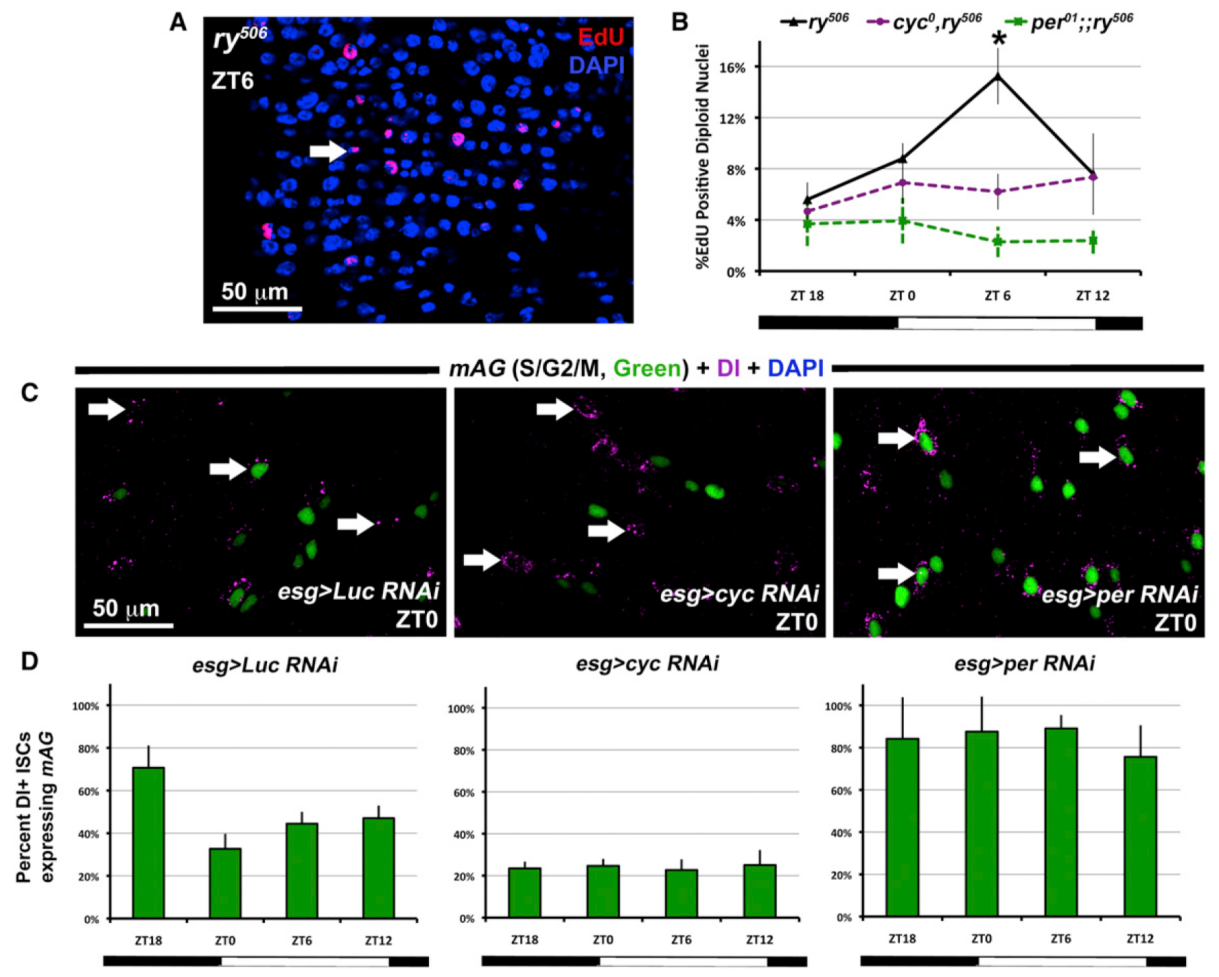
Circadian Rhythms Synchronize Cell-Cycle Phases in ISCs (A and B) Dissected intestines of flies were exposed to the thymidine analog EdU for 45 min to detect S phase cells (red). Control (*ry^506^*) diploid cells in the intestine show a peak of S phase at ZT6, but neither *cyc^0^* nor *per^01^* shows this rhythm (n = 5 guts/genotype/time point, error bars ± SEM, *p < 0.05 at ZT6). (C) The intestines of the FUCCI cell-cycle reporter: *mAG* marks cells in S/G2/M phases, and Dl+ ISCs are indicated with arrows. Analysis is carried out in ISCs (for example, the control *esg > Luc RNAi* indicates esg-Gal4 / UAS-S/G2/M-Green; UAS-Luciferase RNAi / +). (D) Quantification of Dl+ ISCs suggests that most *esg > cyc RNAi* ISCs are negative at all time points, whereas *esg > per RNAi* are positive (green). See also Figures S2 and S3.

The accumulation of mitotic *cyc^0^* ISCs (Figure 2A) suggests that loss of *cyc* throughout the animal causes ISCs to overproliferate or stalls these cells in mitosis. An EdU uptake assay, which measures cells in S phase, revealed that control (*ry^506^*) ISCs show a peak in S phase at ZT6. The *cyc^0^* and *per^01^* mutants do not exhibit any peaks, and *cyc^0^* mutants do not exhibit increased S phase (Figures 3A and 3B). Hence, it is unlikely that *cyc^0^* ISCs overproliferate, and *cyc RNAi* clones also did not show an over-proliferation phenotype (Figure S4).

We applied the FUCCI cell-cycle reporter (Nakajima et al., 2011; Sakaue-Sawano et al., 2008), which accumulates *mAG-Geminin* during S/G2/M phases (*Azami Green* positive), to determine cell-cycle states when circadian rhythms are absent in ISCs. We expressed the FUCCI reporter along with *cyc RNAi* or *per RNAi* with *esg-Gal4*, and identified ISCs using Dl+. The control RNAi lines show a gradual accumulation of S/G2/M-phase-positive ISCs up to ZT18, when these cells divide (Figures 3C and 3D). However, not all ISCs are in S/G2/M phases, indicating that a significant reserve population of ISCs exists at all times. Irrespective of time, nearly all *cyc RNAi* ISCs are S/G2/M phase negative, whereas nearly all *per RNAi* ISCs are positive. Because its loss causes ISCs to accumulate in G1 (or G0), these results suggest that CYC promotes the G1 to S phase transition. Conversely, when PER is lost, movement through G1 is unopposed, but ISCs accumulate after S phase entry without entering mitosis (see Figure 1F). Thus, we propose that the circadian clock regulates the G1 to S phase transition in ISCs following damage.

### The Clock Regulates the Transcription of Hundreds of Genes in the Intestine

**Figure 4:**
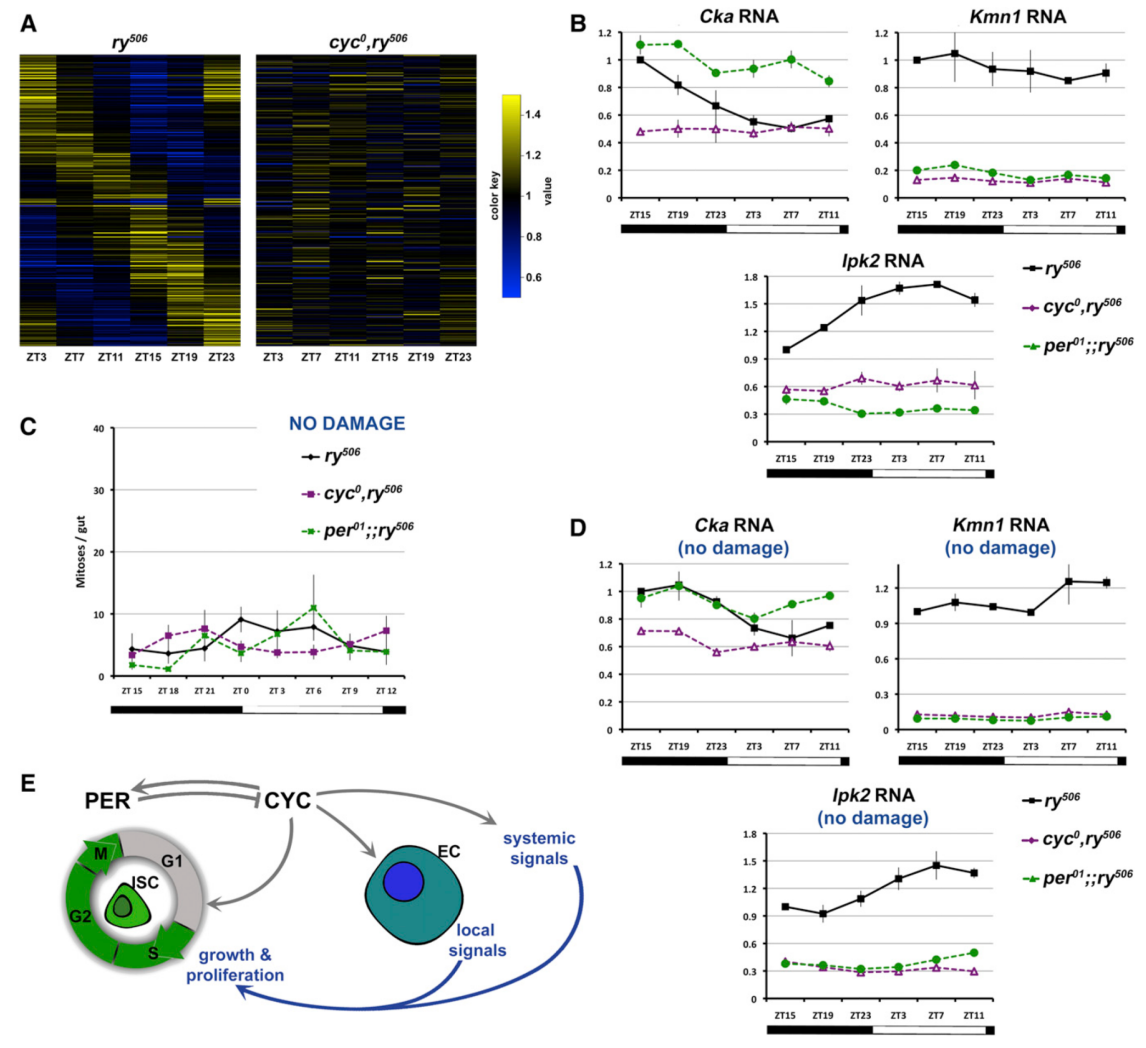
The Clock Regulates the Expression of Diverse Transcripts (A) All genomic transcripts were interrogated for rhythmic expression during regeneration. Heat maps reveal 433 genes with circadian rhythms in *ry^506^* controls but not in *cyc^0^* mutants. (B) *Cka*, *Ipk2*, and *Kmn1* RNA expression (qPCR) in the intestine over 24 hr. *Cka* shows *per*-like rhythms, whereas *Ipk2* exhibits antiphasic rhythms. *Kmn1* displays no circadian rhythmicity but is significantly downregulated in the *cyc^0^* mutant. Graphs are reported as in Figure 1B. (C) Flies maintained in LD conditions on regular media do not show a mitotic peak at ZT0, in contrast to when the intestine is damaged. Under these conditions the mitotic index is similar between *ry^506^* controls and *cyc^0^* or *per^01^* mutants. (D) In the absence of damage, the expression of *Cka* and *Ipk2* (qPCR) is rhythmic, similar to what is observed during regeneration. *Kmn1* (qPCR) also shows lower expression both before and after damage. (E) A model of how the clock synchronizes ISC division: CYC is important for the transition through G1, and the clock also initiates systemic signals and local niche signals originating from ECs. Together, these signals activate ISC divisions, most likely through nonautonomous mechanisms. See also Figures S2, S3, and S5.

More than 10% of all mammalian genes are regulated in a circadian fashion (Panda et al., 2002), and components of the clock directly regulate transcription in a tissue-specific manner (Abruzzi et al., 2011; Akhtar et al., 2002), suggesting that a tremendous variety of cell states are outcomes of circadian processes. Since *per* RNA and protein oscillate in the midgut, and *per* was identified in our screen, we performed genome-wide expression analysis on *ry^506^* control intestines and *cyc^0^* mutants over 24 hr following damage (Figure 4A; Tables S1, S2, and S3). We reasoned that clock target genes would show 24 hr rhythms and would be perturbed if CLK/CYC were disrupted. We found that 433 genes were rhythmic in controls, like *per*, but arrhythmic in *cyc^0^*, indicating that they are under clock regulation in this tissue (Table S1). For instance, *Connector of kinase to AP-1* (*Cka*), a scaffold protein required for signal transduction of the JNK stress-response pathway (Chen et al., 2002), peaks at ZT15 (Figure 4B). Direct CLK/CYC targets would be expected to be strongly reduced in *cyc^0^* mutants, yet only 21 of 433 genes (including *per* and *tim*) fit this profile (Table S2); hence, most rhythmic genes are likely to be indirectly regulated. Two hundred rhythmic genes showed the opposite phase to that of *per*, suggesting they are regulated by the transcription factors *vrille* or *Pdp1*, which are part of the clock and together generate antiphasic transcript rhythms that peak in the early day (Hardin, 2011;Table S1). One of these, *Ipk2*, is an inositol phosphate kinase and a positive regulator of Jak/STAT signaling (Müller et al., 2005), a pathway that is critical during intestinal regeneration (Figure 4B). Another one of these genes, *bazooka*, was recently reported to polarize ISCs (Goulas et al., 2012), suggesting that the clock also regulates cell polarity. An additional 205 genes showed low expression in *cyc^0^* mutants but did not display rhythms (Table S3). This includes *Kmn1*, which enables chromosome segregation during anaphase (Venkei et al., 2011), suggesting that mitosis could be disrupted (Figure 4B). Overall, a great diversity of intestinal transcripts are thus influenced by the clock.

## DISCUSSION

Circadian pathway mutants are viable and their cells readily proliferate during development. Unlike other tissues (Abruzzi et al., 2011; Borgs et al., 2009), cell-cycle regulators do not seem to be clock targets in the intestine (Table S1). Although they are readily detected, neither cyclins nor regulators such as *Wee1* (Matsuo et al., 2003) exhibit circadian rhythms in this tissue. In the absence of acute damage, clock mutant ISCs divide normally (Figure 4C) and have no ISC-autonomous phenotypes (Figure S4). So it is quite surprising that PER and CYC are critical for adult ISC division during regeneration.

The ISC-autonomous phenotypes that occur during regeneration are modest compared with those that arise when the clock is disrupted systemically or in all ISCs/ECs by RNAi. This suggests that the clock predominantly regulates nonautonomous functions and may be involved in the synchronization of cell states across this tissue during the damage response. Indeed, because *esg-Gal4* is expressed in both ISCs and their immediate progeny (the EBs) for some time while they differentiate, it is possible that the clock regulates EB-to-ISC signaling. Intriguingly, disruption of the circadian clock in different cells leads to the accumulation of ISCs in different cell states; for instance, the *cyc^0^* mutant stalls during mitosis when CYC is absent systemically (Figure 2A), whereas it stalls during G1 if CYC is depleted in all ISCs (Figures 3C and 3D). This G1 lag explains why *cyc RNAi* ISCs show reduced mitoses compared with the *cyc^0^* mutant; however, given that the mechanisms underlying these processes are unresolved, it is possible that these differences are due to genetic background. At present, we thus conclude that rhythmic cell proliferation normally occurs in the damaged intestine and that this is dependent on the clock. We also note that forced expression of *per* or *cyc* in ISCs is able to partially restore rhythmic divisions in their respective mutant backgrounds (Figures 1E and 2A), whereas disruption of these genes in only ECs perturbs ISC rhythmic division (Figures 1G and 2F). This highlights the complexity of clock-regulated processes and suggests that desynchrony between ISCs and their surrounding cells (Figures S1G and S1H) can have different outcomes.

Circadian rhythms occur in many intertwined processes, including metabolism (Sahar and Sassone-Corsi, 2009), post-transcriptional regulation (Koike et al., 2012), and oxidation-reduction cycles (O’Neill and Reddy, 2011). The rhythmic expression of *Cka*, which brings together kinases and transcription factors to transduce JNK signal (Chen et al., 2002), and *Ipk2*, which may boost the activity of cytokines involved in regeneration (Müller et al., 2005), suggests that the clock sensitizes the intestine to engage the regenerative response at specific times. For instance, several of the genes that exhibit circadian rhythms during regeneration also show these rhythms prior to damage (Figure 4D). An emergent function of the clock could be to coordinate stem cell states according to either local niche signals or systemic signals, each of which would be under autonomous circadian control (Figure 4E).

Although *per* mutation increases cancer incidence (Borgs et al., 2009; Fu et al., 2002; Wood et al., 2008) and cancer cell proliferation (Borgs et al., 2009; Janich et al., 2011), our work suggests it is not simply a tumor suppressor. Recently, the circadian clock was shown to influence mammalian blood and hair stem cell biology (Janich et al., 2011; Méndez-Ferrer et al., 2008). In particular, hair stem cells are strikingly heterogenous in their circadian rhythm activity (Janich et al., 2011), for unknown reasons. The coordination of proliferation, by synchronizing internal with external rhythms, may thus represent an important difference between normal stem cells and neoplastic cells.

## EXPERIMENTAL PROCEDURES

Animals were maintained at 25°C under LD conditions and damaged by being fed 5% w/v DSS (MP Biomedicals) or 25 μg/mL Bleocin (Calbiochem). The flies were maintained under LD conditions as before, except for experiments in which the light conditions were changed to complete darkness or complete light. Female flies < 14 days of age were used in all experiments, with the exception of the mosaic analysis. The following *Drosophila* lines were used:

OreR
ry^506^
y, w
cyc^0^, ry^506^
per^01^;; ry^506^
per^01^; tim^0^; ry^506^
per^01^;; cyc^0^, ry^506^
y, w; tim^0^
UAS-per16
UAS-cyc6
esg-Gal4
esg-Gal4, UAS-eGFP, tub-Gal80^TS^
myo1A-Gal4
tim-Gal4
hsFlp, FRT19A, tub-Gal80; act < y+ < Gal4, UAS-GFP / CyO
hsFlp; act > CD2 > Gal4, UAS-nlsGFP / Cyo
w; UAS-dcr2 (II)
w; UAS-dcr2 (III)
UAS-S/G2/M-Green / CyO
*cyc RNAi* (National Institute of Genetics #8727R-1, Mishima, Shizuoka, Japan)
*per RNAi* (TRiP #JF01226, Harvard Medical School, Boston, USA).
*Luc RNAi* (TRiP #JF01355, Harvard Medical School, Boston, USA).

Full details regarding the procedures are provided in Extended Experimental Procedures.

## Supplementary Material

Supplementary Table 1

Supplementary Table 2

Supplementary Table 3

Supplementary Material
