# Supplementary material for: The Circadian Clock Gates the Intestinal Stem Cell Regenerative State
Source: Cell Rep. Author manuscript; Available in PMC 2014 Apr 25. (PMC3982394; doi:10.1016/j.celrep.2013.03.016)
Supplement: Supplementary Material [file NIHMS569196-supplement-04.pdf]

# Supplemental Information

## EXTENDED EXPERIMENTAL PROCEDURES

### Housing
Animals were maintained at 25°C, 12 hr light and 12 hr dark (LD) conditions, under constant humidity. For damage, a 5% w/v sucrose (Sigma) + water solution containing either 5% w/v DSS (MP Biomedicals) or 25 ug/mL Bleocin (Calbiochem) were applied for 2 days prior to timeseries analysis (2–3 days total exposure). Chemicals were refreshed daily, and flies were maintained on LD conditions as before, with the exception of experiments where the lights were changed to complete darkness or complete light (see Figures S1 and S5 for experimental schematics). Female flies of < 14 days of age were used in all experiments, except in the mosaic analysis where guts were obtained from flies 23 days following clone induction.

### Dissection and Staining
Guts were dissected in 1X PBS (GIBCO) and fixed in 4% paraformaldehyde (Electron Microscopy Sciences) diluted with 1X PBS. Samples were washed 3X with PBS, then blocked for 30 min in 1X PBS, 1% BSA (Sigma), 0.2% Triton X-100 (Sigma). The following antibodies were used: Rabbit anti-Period, Mouse anti-Delta (Developmental Studies Hybridoma Bank), Mouse anti-Prospero (Developmental Studies Hybridoma Bank), Mouse anti-Fibrillarin (Encore Biotechnology), Rabbit anti-phospho-Histone3 (Millipore). Samples were washed 3X with PBS, and stained with secondary antibodies: Donkey anti-mouse Alexa 555 (Molecular Probes), Donkey anti-mouse Alexa 647 (Molecular Probes), Goat anti-rabbit Alexa 488 (Molecular Probes), Donkey anti-rabbit Alexa 555 (Molecular Probes). EdU exposure was carried out for 45 min on dissected guts according to the manufacturer's instructions, labeling was also performed according to the Click-iT EdU Alexa Fluor 555 Imaging Kit (Invitrogen) instructions. All samples were counterstained with DAPI (Molecular Probes) and mounted using Vectashield (Vector). For brain dissection, 2–5 day post-eclosion flies were entrained 3 days under LD conditions, and were stained as described in Zhang et al. (Zhang et al., 2010).

### RNAi Screen
We performed a genetic screen for transcription factors required in *Drosophila* ISCs during regeneration, by expressing *UAS-RNAi* constructs (Ni et al., 2009) to suppress transcription factors/regulators, using a temperature-inducible *esg-Gal4, tub-Gal80TS* driver. Flies were raised at 18°C to prevent the expression of UAS-RNAi, then, at 2–5 days post-eclosion, shifted to 29°C for 8 days to induce expression in ISCs. Flies were exposed to 5% DSS for an additional 3 days, and guts were dissected and stained for phospho-Histone3 as described above. Total mitoses per gut were scored for each RNAi and compared to Luc RNAi controls, whose division is increased 10-20 fold from baseline in the presence of DSS. Two separate RNAi constructs for *per* were recovered as strong hits in the screen, suppressing division by at least 80%.

### RNA Purification and qRT-PCR
15–20 midguts from each genotype were collected in RNAlater reagent (QIAGEN; n = 3 biological replicates). mRNA was isolated using a Oligotex Direct mRNA Mini Kit and/or RNA using the RNEasy Mini Kit (QIAGEN), and the iScript cDNA Synthesis Kit (Biorad) was used to transcribe cDNA. qPCR was then carried out on a CFX96 Real-Time System / C1000 Thermal Cycler (Biorad) with iQ SYBR Green Supermix (Biorad). Expression was normalized to *GAPDH* control transcript, then normalized relative to the appropriate control at ZT15. qPCR primers:

GAPDH1-F: CCAATGTCTCCGTTGTGGA
GAPDH1-R: TCGGTGTAGCCCAGGATT
per-F: TCATCCAGAACGGTTGCTACG
per-R: CCTGAAAGACGCGATGGTGT
tim-F: CCAGCATTCATTCCAAGCAG
tim-R: GCGTGGCAAACTGTGTTATG
Cka-F: aaggatatgctcaccgagga
Cka-R: gccatcagattcgttattcacc
Kmn1-F: tcgctatgaagcaagcacttt
Kmn1-R: cctcgtcctcctgacagcta
Ipk2-F: attgccgccttcagaggt
Ipk2-R: atgacggcgcggtagtagt

### Microarray Analysis
n = 20 midguts, in duplicate, were collected every 4h over 24h following Bleocin damage. RNA was purified as above, then processed and hybridized on Affymetrix *Drosophila* Genome 2.0 chips, according to the manufacturer's instructions, at the Microarray Core, Dana-Farber Cancer Institute. Data were normalized and analyzed as described in Xu et al. (2011).

## Survival Assays

Flies were maintained under light/dark conditions as above. Flies were fed either 5% or 10% w/v DSS (MP Biomedicals) or 25 ug/mL Bleocin (Calbiochem), mixed in a solution of 5% w/v sucrose (Sigma). Approximately 15 flies were loaded per vial (n = 3 vials per genotype), and solutions were refreshed daily. Survival was scored each day and assays were performed on 2 separate occasions.

## CAFÉ and Blue-Dye Assays

Flies were maintained under light/dark conditions as above. Blue dye assay was performed as reported in Xu et al. (2008) For the CAFÉ assay, 5 mL of 1% *Drosophila* Type II Agar (Apex BioResearch Products) was solidified in one vial to provide hydration. 5-10 flies were loaded into each of these vials and a capillary of feeding solution (5% w/v sucrose and 2.5% w/v yeast extract) was presented in each. Flies were maintained for one day, to acclimatize to feeding from capillaries, before the start of the assay. Capillaries were refreshed once every 3 hr, and the solution levels consumed in each capillary noted.

## Behavior Assay

Adult flies (2–5 days old) were entrained for 3 days under LD (500 lux intensity), and then released into constant darkness for at least 5 days. Single fly locomotor activity was measured with TriKinetics Activity Monitors (Waltham, MA) in I36-LL Percival Incubators. Data analysis was performed using the FAAS-X software (Grima et al., 2002).

## Mosaic Analysis

Flies were maintained on a 12–12 light/dark cycle, as above, and transferred to fresh media every 1–2 days. For MARCM clones two 1 hr 37°C heat shocks were applied at 2–3 days following eclosion, while for RNAi clones one 12 min 37°C heat shock was applied at 2–3 days following eclosion. A region of leaky GFP expression was noted in the RNAi clone stock, and this was omitted from these analyses. Clones were scored as clusters of 2 or more directly adjacent cells.

## Imaging and Data analysis

Confocal microscopy was done using the Zeiss LSM 780 with the Zen LSM software package (Zeiss). Images were processed in Photoshop CS5.1 (Adobe). The percentage of cells was calculated by determining the number of (+) cells of interest divided by the total DAPI+ nuclei in one FOV from posterior midgut using a 40X objective. ~300–400 cells were scored per FOV per midgut, and > 5 midguts were analyzed per genotype. The same approach was used to quantify the percentage of (+) cells using the FUCCI cell cycle reporter and EdU, but in these cases, the total was that of the total Dl+ or total diploid cell nuclei present, respectively. Statistics (either paired or unpaired t tests, or ANOVA with Dunnett's post-test to compare to controls, as appropriate) were carried out using Graphpad Prism 5.0.

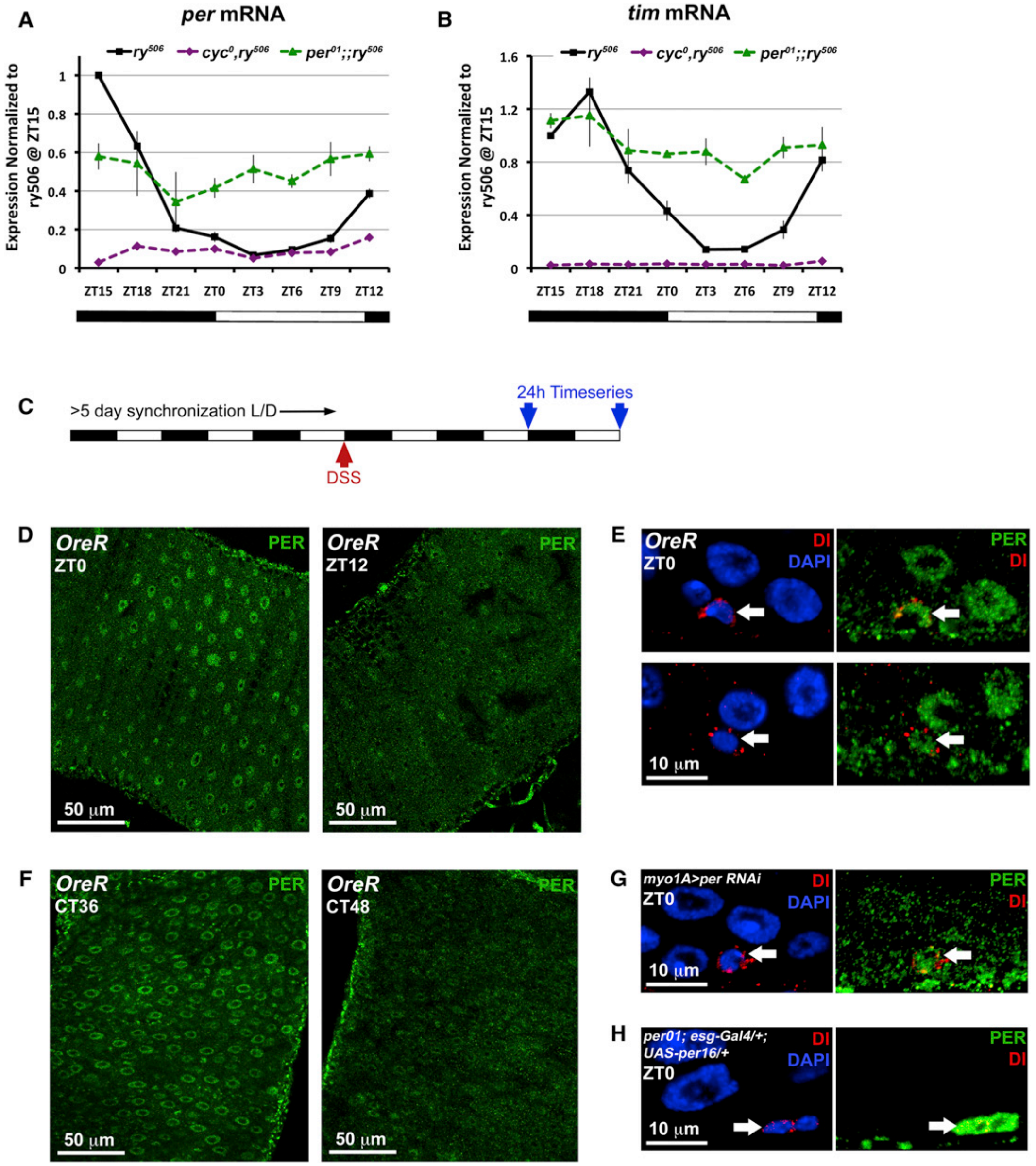


**Figure S1. PER/TIM Expression in the Gut, Related to Figures 1 and 2**

(A) *per* RNA expression (qPCR) over Zeitgeber Time (ZT) normally shows circadian rhythms in the intestine (*ry*$^{506}$ control), which are absent in *per*$^{01}$ (*ry*$^{506}$ and *per*$^{01}$ data are the same as in Figure 1B). The *per*$^{01}$ mutation is a nonsense mutation at exon 4 that produces a truncated non-functional product. *per* expression is very low in *cyc*$^{0}$, because *per* is a direct transcriptional target of CLK/CYC.

(B) *tim* is also a clock target and fluctuates in the same manner as *per*. *tim* RNA remains high in the *per*$^{01}$ mutant (qPCR), and the *cyc*$^{0}$ mutant shows very low *tim* RNA level. As *per/tim* are transcribed by CLK/CYC they increase in level and eventually repress their own transcription. Degradation of PER/TIM eventually frees CLK/CYC, thus completing the feedback loop. Graphs a-b show average of 2 separate experiments (n = 15 guts/genotype/time point, $p < 0.05$ by ANOVA, expression normalized to *ry*$^{506}$ ZT15, relative to *GAPDH* control RNA, error bars ± SEM).

(C) Schematic shows synchronization of flies on 12-12 LD cycle (for at least 3 days), followed by DSS (or Bleocin) application and 24 hr analysis 2 days later. See Extended Experimental Procedures for full description.

(D) PER staining (green) shows nuclear accumulation in intestinal epithelial cells in the morning (ZT0) versus the evening (ZT12), ECs can be recognized by their large polyploid nuclei.

(E) At ZT0, PER protein is expressed in ISCs (arrows) labeled with Delta (Dl, red).

(F) PER staining under DD conditions similarly shows accumulation and reduction over 24h rhythms.

(G) PER protein does not accumulate in ECs at ZT0 when *per RNAi* is expressed in ECs using *myo1A-Gal4*.

(H) Similarly PER is not present in ECs, but is present in ISCs and EBs when the PER rescue construct is forcibly expressed using *esg-Gal4* in the *per*$^{01}$ mutant background. Note that PER is present at much higher levels using the rescue construct (compare with Figure 1D and S1E). This suggests the ISCs and ECs are desynchronized in these two experiments.

Cell PRESS
Open ACCESS

A

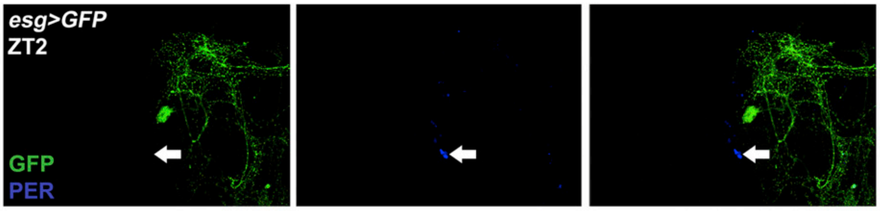


B

| Genotype (males) | Number | Rhythmicity | Tau±Sem | Pwr±Sem |
|---|---|---|---|---|
| *myo1A-Gal4/+; Per RNAi/+* | 14 | 78.6% | 24.2±0.07 | 54.2±5.23 |
| *esg-Gal4/+; Per RNAi/+* | 15 | 93.3% | 24.5±0.06 | 64.0±3.79 |
| *Tim-Gal4/+; Per RNAi/+* | 8 | 100% | 27.5±0.40 | 56.3±5.17 |
| *Tim-Gal4, UAS-dcr2/+; Per RNAi/+* | 16 | 0% | | |
| *myo1A-Gal4/+; Cyc RNAi/+* | 14 | 78.6% | 24.6±0.06 | 41.6±5.92 |
| *esg-Gal4/+; Cyc RNAi/+* | 15 | 86.7% | 25.1±0.13 | 60.1±4.59 |
| *Tim-Gal4/+; Cyc RNAi/+* | 8 | 0% | | |
| *Tim-Gal4, UAS-dcr2/+; Cyc RNAi/+* | 12 | 0% | | |
| *myo1A-Gal4/+; Luc RNAi/+* | 15 | 86.7% | 24.0±0.13 | 49.2±5.21 |
| *esg-Gal4/+; Luc RNAi/+* | 9 | 55.6% | 24.9±0.08 | 52.7±10.16 |
| *Tim-Gal4/+; Luc RNAi/+* | 8 | 100% | 25.1±0.10 | 53.4±8.05 |
| $w^{1118}$ | 9 | 100% | 24.1±0.11 | 65.1±8.66 |

| Genotype (males) | Number | Rhythmicity | Tau±Sem | Pwr±Sem |
|---|---|---|---|---|
| $per^{01}$;; *UAS-per16* | 21 | 0% | | |
| $per^{01}$; *myo1A-Gal4/+; UAS-per16/+* | 20 | 0% | | |
| $per^{01}$; *esg-Gal4/+; UAS-per16/+* | 15 | 0% | | |
| $per^{01}$; *Tim-Gal4/+; UAS-per16/+* | 19 | 57.9% | 24.3±0.20 | 45.6±4.62 |
| $w^{1118}$ | 8 | 100% | 23.7±0.04 | 104.5±9.18 |

C

| Genotype (females) | Number | Rhythmicity | Tau±Sem | Pwr±Sem |
|---|---|---|---|---|
| *myo1A-Gal4/+; Per RNAi/UAS-dcr2* | 20 | 100% | 24.1±0.07 | 64.5±4.20 |
| *esg-Gal4/+; Per RNAi/UAS-dcr2* | 31 | 71.0% | 25.1±0.10 | 35.3±8.45 |
| *Tim-Gal4/+; Per RNAi/UAS-dcr2* | 24 | 0% | | |
| *myo1A-Gal4/+; Cyc RNAi/UAS-dcr2* | 28 | 92.9% | 24.6±0.09 | 59.3±4.81 |
| *esg-Gal4/+; Cyc RNAi/UAS-dcr2* | 36 | 66.7% | 25.8±0.12 | 36.1±3.27 |
| *Tim-Gal4/+; Cyc RNAi/UAS-dcr2* | 24 | 0% | | |
| *myo1A-Gal4/+; Luc RNAi/UAS-dcr2* | 23 | 91.3% | 23.9±0.11 | 55.3±5.25 |
| *esg-Gal4/+; Luc RNAi/UAS-dcr2* | 30 | 33.3% | 24.3±0.13 | 35.1±6.20 |
| *Tim-Gal4/+; Luc RNAi/UAS-dcr2* | 24 | 58.3% | 24.5±0.20 | 26.5±4.05 |

| Genotype (females) | Number | Rhythmicity | Tau±Sem | Pwr±Sem |
|---|---|---|---|---|
| $per^{01}$;; *UAS-per16* | 16 | 0% | | |
| $per^{01}$; *esg-Gal4/+; UAS-per16/+* | 16 | 0% | | |

**Figure S2. Validation of RNAi and Rescue Reagents, Related to Figures 1, 2, 3, and 4**

(A) The *esg-Gal4* driver (*esg* > *GFP* is *esg-Gal4, UAS-GFP*) is expressed in several neurons in the brain, but it is not expressed in pacemaker neurons stained for PER protein (blue). Image shows a single confocal plane. Similarly, *myo1A-Gal4* was not colocalized with pacemaker neurons (not shown).

(B and C) Tables show periods of activity in male and female flies in constant darkness, following light/dark synchronization. Flies normally exhibit characteristic peaks of activity in the early morning and late evening which oscillate in a circadian manner (see for instance $w^{1118}$ and *Luc RNAi* controls). Expression of *UAS-per RNAi* in pacemaker neurons using *tim-Gal4* completely disrupts behavior rhythms with *UAS-dcr2*, and increases the period of rhythms without *UAS-dcr2*. Similarly, *UAS-cyc RNAi* in pacemaker neurons completely disrupts behavior rhythms with and without *UAS-dcr2*. In contrast behavior rhythms persist when the same constructs are expressed in ISCs using *esg-Gal4*, or in ECs using *myo1A-Gal4*. We note, however, that the period of behavior rhythms is slightly (~1h) longer than 24h in *esg* > *Luc RNAi* (24.9h for males, 24.3 for females), esg > *cyc RNAi* (25.1 for males, 25.8 for females), and *esg* > *per RNAi* (24.5h for males, 25.1 for females). We further tested the possibility that the *esg-Gal4* and *myo1A-Gal4* drivers were expressed in the brain, by using them to express a rescue construct, *UAS-per16*, in the $per^{01}$ mutant background. $per^{01}$ flies are completely arrhythmic, but *tim-Gal4* driven expression of *UAS-per16* effectively restores behavior rhythms. In contrast, neither *esg-Gal4* or *myo1A-Gal4* restore rhythms to $per^{01}$. The *tubulin-Gal4* driver knocks down *cyc* mRNA (*tub-Gal4/+;UAS-cyc RNAi/+*) to 20% of wild-type *cyc* levels at ZT3, and *per* mRNA (*tub-Gal4/+;UAS-per RNAi/+*) to 25% of wild-type *per* levels at ZT15 (peak of *per* expression). These data support the use of the *esg-Gal4* and *myo1A-Gal4* drivers and RNAi constructs to disrupt circadian rhythms in ISCs or ECs, respectively, without affecting circadian behavior.

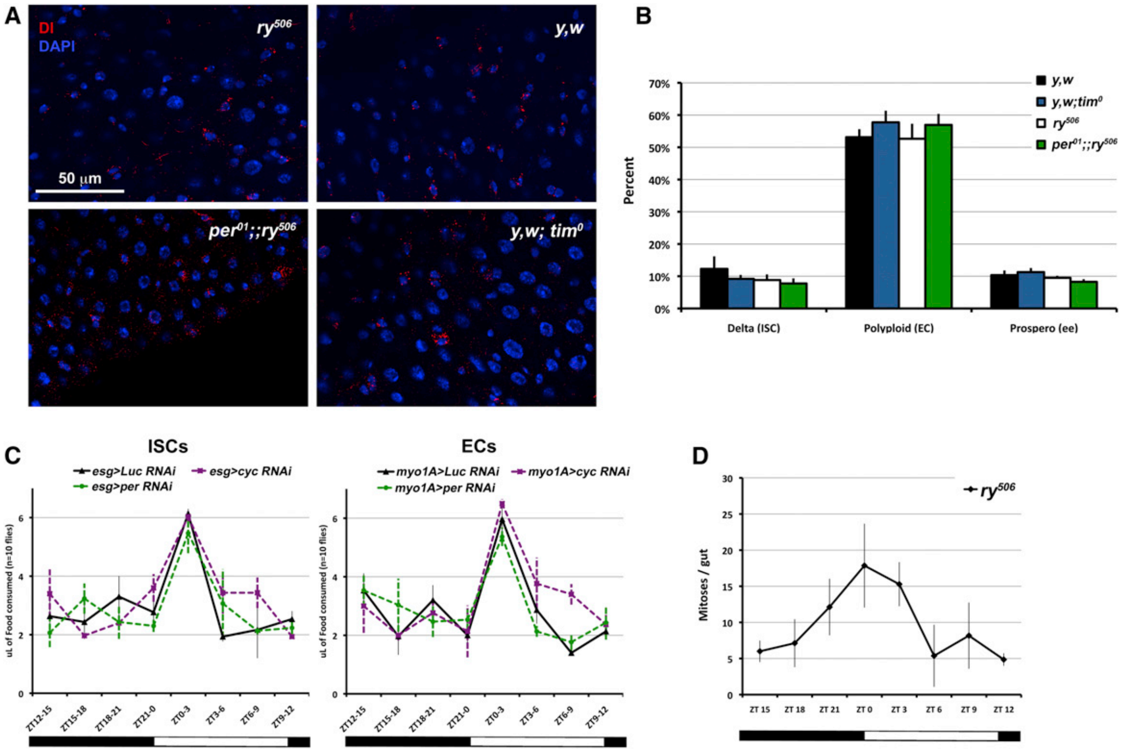


**Figure S3. Circadian Clock Mutants Show No Obvious Intestinal Cell Deficiencies, and Rhythmic Mitoses in the Intestine Are Not Dependent on Feeding, Related to Figures 1, 2, 3, and 4**

(A) ISCs, labeled for Delta (Dl, red) are evident in both *per*$^{01}$ and *tim*$^{0}$ mutant intestines.

(B) The proportion of ISCs (%Delta+), ECs (%polyploid nuclei), or ees (%Prospero+) does not significantly vary between *per*$^{01}$ and *tim*$^{0}$ mutant, and *ry*$^{506}$ or *y,w* control intestines. *cyc*$^{0}$ mutant intestines showed a similar proportion of these cell types (not shown).

(C) If perturbation of circadian rhythms affected feeding, the reduction and arrhythmic intestinal mitoses in the *per*$^{01}$ mutant or the *esg > per RNAi* could be simply a result of insufficient uptake of DSS. It has been previously shown that circadian clock genes regulate the timing of feeding rhythms, but that mutants do not feed less over a 24h period (Xu et al., 2008). Although we did not observe any mitotic rhythms in the intestine of the mutants tested, the CAFÉ assay, was applied to test the amount of food consumed by flies over 24 hr. The volume of media consumed was measured at 3h intervals, where knockdown of CYC or PER in either the ISCs or ECs had no effect on feeding. Because mitoses peak at ZT0, and the highest level of food consumed is from ZT0-ZT3, this suggests that mitoses peak before feeding levels. In addition, the amount of food consumed over the entire 24h period was equivalent between all of these genotypes (not shown). For instance, *esg > per RNAi* shows very low and arrhythmic mitoses at all time points (Figure 1F), but feeds equivalently to the *esg > Luc RNAi* control, whose mitoses are higher and rhythmic over the same time points. Finally, we tested feeding levels using a blue-dye uptake assay and found that *ry*$^{506}$, *per*$^{01}$, *cyc*$^{0}$ and *tim*$^{0}$ animals fed equivalently over 24 hr (not shown).

(D) Flies were entrained on LD conditions as before but food was presented from ZT12-ZT0 only, and flies were shifted onto 5% agar/$H_2$0 from ZT0-ZT12. Following 2 days DSS exposure (ZT12-ZT0 only), mitotic rhythms still peak at ZT0 further suggesting that ISC division is independent from the timing of DSS consumption. We note, however, that mitoses are reduced in these conditions, perhaps as a result of the restricted availability of DSS.

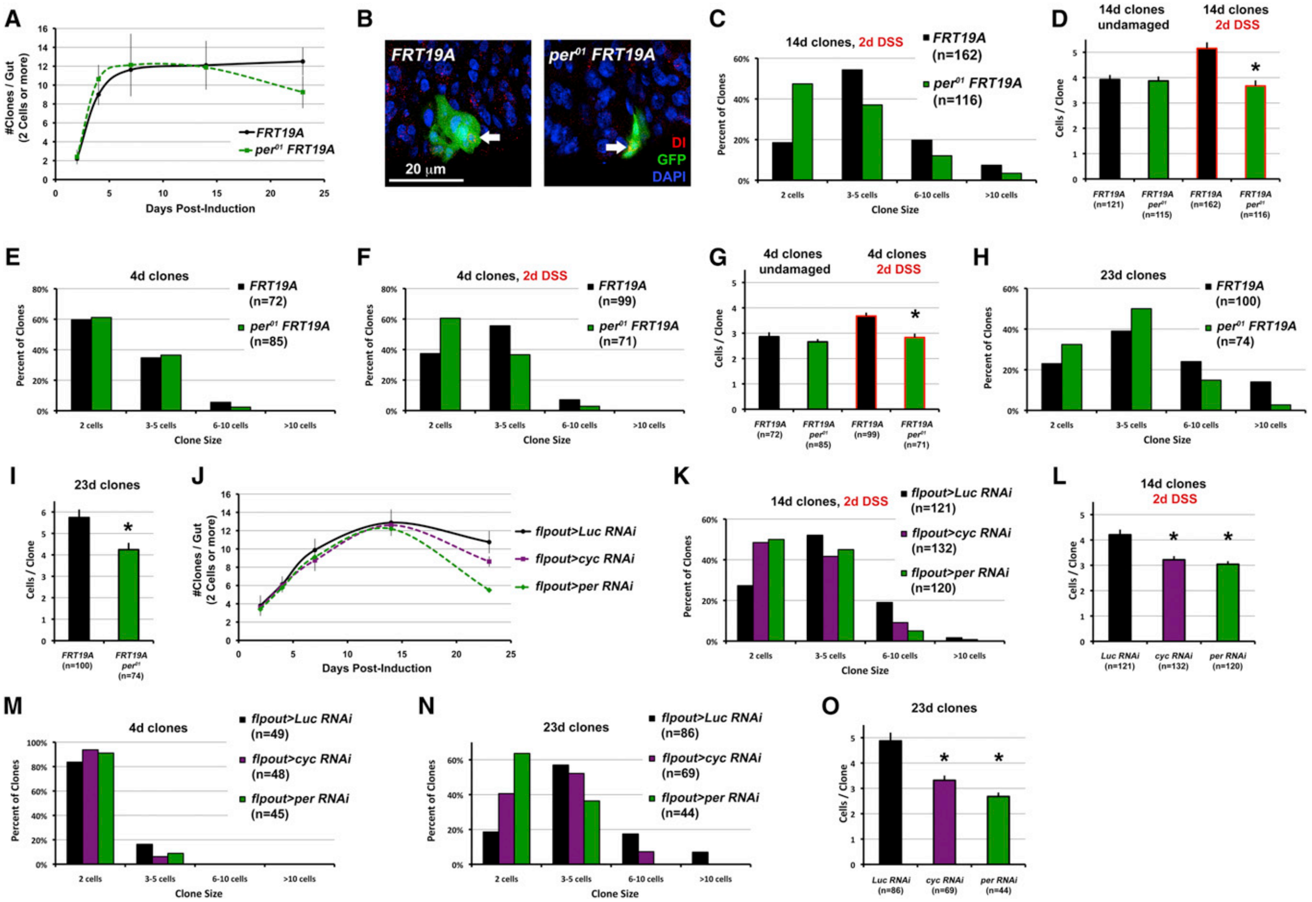


**Figure S4. ISC-Autonomous Proliferation Is Decreased when CYC or PER Is Lost, Related to Results and Discussion**

(A) The frequency of proliferating clones ($\geq$2 cells in size) in the whole gut was measured over 23 days following clonal induction (n = 10 guts/genotype/time point, error bars $\pm$ SEM).

(B and C) 14 days following induction, control (*FRT19A* is *FRT19A/hsFlp,FRT19A,Tub-Gal80; act > y+ > Gal4, UAS-GFP/+*) clones, under 2 days DSS damage, are larger than *per* mutant clones (*per$^{01}$ FRT19A* is *per$^{01}$, FRT19A/hsFlp,FRT19A,Tub-Gal80; act > y+ > Gal4, UAS-GFP/+*). Note that Dl+ cells are present in *per$^{01}$* clones, indicating that the ISCs are not lost. *per$^{01}$* clones show a different distribution of sizes, brackets indicate number of clones examined.

(D) 14 days following induction, *per$^{01}$* are the same size as wild-type, however upon damage *per$^{01}$* clones are slightly but significantly smaller. Damaged clones are same as those reported in Figure S4C. Brackets indicate number of clones examined, *significance by t test ($p < 0.05$).

(E) At 4 days, the size of *per$^{01}$* clones is similar to controls. Brackets indicate number clones examined.

(F) 4 day *per$^{01}$* clones, exposed to DSS for 2 days, show a different distribution of sizes. Brackets indicate number of clones examined.

(G) 4 days following induction, *per$^{01}$* are the same as wild-type, however upon damage *per$^{01}$* clones are slightly but significantly smaller. Damaged clones are the same as those reported in Figure S4F. Brackets indicate number of clones examined, *significance by t test ($p < 0.05$).

(H) At 23 days, in the absence of damage, *per$^{01}$* clones show a different distribution of sizes. Brackets indicate number of clones examined.

(I) The average size of *per$^{01}$* clones at 23 days following induction, even in the absence of damage, is significantly smaller than controls. Clones are the same as those in Figure S4H. Brackets indicate number of clones examined, *significance by t test ($p < 0.05$).

(J) The same assay was undertaken using RNAi clones (for instance the control *flpout > Luc RNAi* refers to *hsFlp/+; act > CD2 > Gal4,UAS-nlsGFP/UAS-dcr2; UAS-Luciferase RNAi/+*). All genotypes are only different in the RNAi transgene on chromosome 3 (n = 10 guts/genotype/time point, error bars $\pm$ SEM).

(K) 14 day *cyc RNAi* and *per RNAi* clones, exposed to DSS for 2 days, show a different distribution of sizes. Brackets indicate number of clones examined.

(L) *cyc RNAi* and *per RNAi* clones are slightly but significantly smaller. Clones are the same as those reported in Figure S4K. Brackets indicate number of clones examined, *significance by t test ($p < 0.05$).

(M) At 4 days, the size of *cyc RNAi* and *per RNAi* clones is similar to controls. Brackets indicate number clones examined.

(N) At 23 days, with no damage, both *cyc RNAi* and *per RNAi* clones show different size distributions compared to *Luc RNAi* controls. Brackets indicate number clones examined.

(O) Both *cyc RNAi* and *per RNAi* 23 day clones are slightly but significantly smaller. Clones are the same as those reported in Figure S4N. Brackets indicate number of clones examined, *significance by t test ($p < 0.05$).

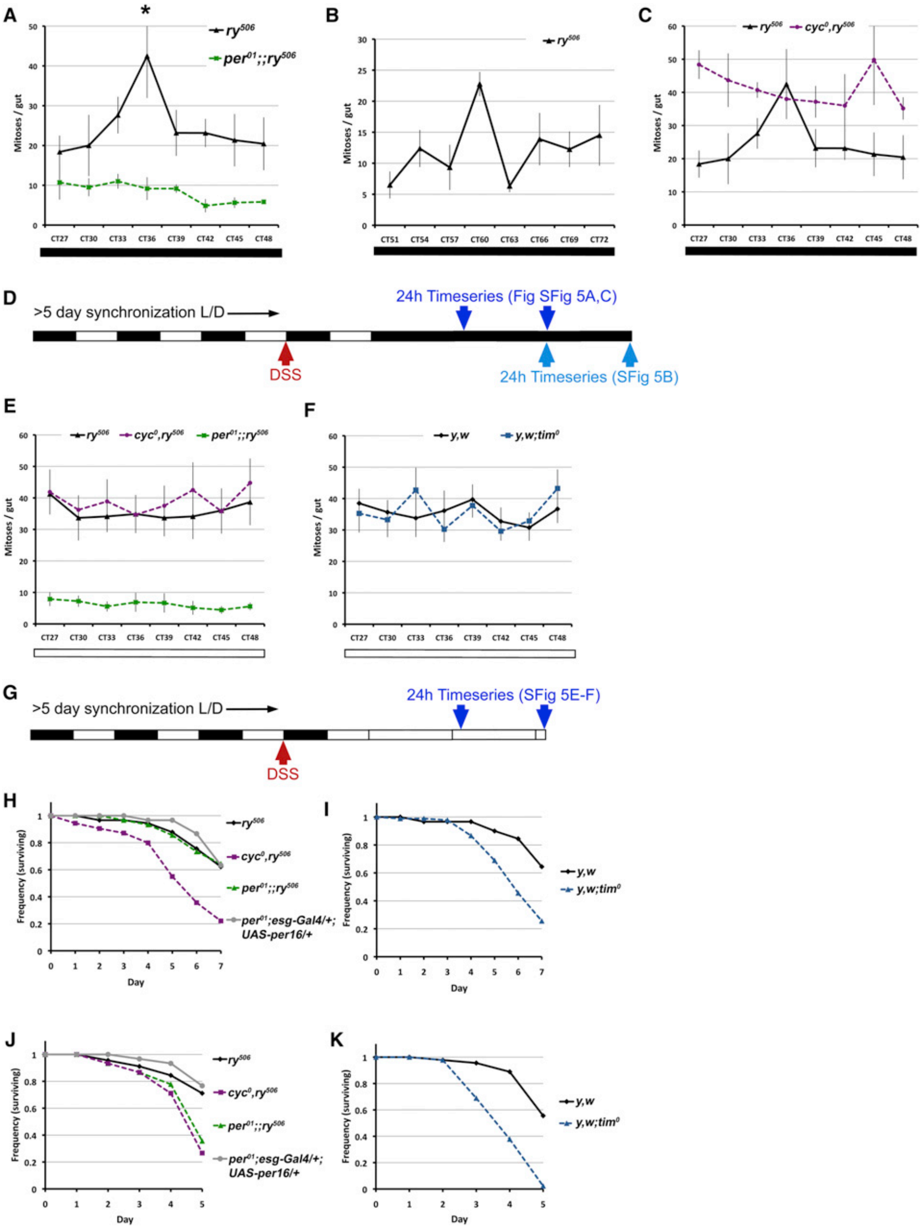


**Figure S5. Mitotic Rhythms Are True Circadian Rhythms and Are Required for Survival following Gut Damage, Related to Figures 1, 2, and 4**

(A) If lights are turned off, after light/dark synchronization (see schematic below), rhythms in mitoses persist 2 days later in *ry*$^{506}$ but not in *per*$^{01}$.

(B) If lights are turned off, after light/dark synchronization, rhythms in mitoses also persist 3 days later in *ry*$^{506}$. The peak in mitoses seen at 2–3 days DD thus repeats itself 24h later, at 3–4 days, in the absence of light cues.

(C) *cyc*$^{0}$ mutant intestines were examined when flies were shifted from 12-12 light/dark conditions to complete darkness (as in schematic below). The circadian rhythms in controls are perpetuated in these conditions (*ry*$^{506}$ data is taken from Figure S5A), but *cyc*$^{0}$ intestines remain arrhythmic and at same levels as in Figure 2A. Graphs show n = 10 guts/genotype/time point (error bars ± SEM).

(D) Schematic shows synchronization of flies on 12-12 Light/Dark cycle (for at least 3 days), followed by DSS application, shifting to complete darkness, and 24 hr analysis 2 days later. See Extended Experimental Procedures for full description.

(E and F) When light/dark synchronized flies are exposed to constant light (as in schematic below), mitotic rhythms in the intestine are no longer observed (n = 10 guts/genotype/time point, error bars ± SEM). Neither *ry*$^{506}$ nor *y,w* control flies exhibit peaks of mitoses at ZT0 under constant light (compare with Figures 1E, 2A, and 2B), rather these show high mitoses at all times.

(G) Schematic shows synchronization of flies on 12-12 Light/Dark cycle (for at least 3 days), followed by DSS application, shifting to complete light, and 24 hr analysis 2 days later. See Extended Experimental Procedures for full description.

(H and I) Survival of circadian mutant animals compared to their respective controls on 5% DSS (black lines). *cyc*$^{0}$ and *tim*$^{0}$ mutants show reduced survival on 5% DSS.

(J and K) Survival assays of same genotypes on 10% DSS, where *cyc*$^{0}$, *tim*$^{0}$, and *per*$^{01}$ mutants show reduced survival as they do on Bleocin (Figures 2H and 2I) Graphs show representative experiments (n = 3 vials/15 flies per vial, genotypes are as above).
